# Bioactive compounds and techno-functional properties of high-fiber co-products of the cacao agro-industrial chain

**DOI:** 10.1016/j.heliyon.2021.e06799

**Published:** 2021-04-16

**Authors:** Johannes Delgado-Ospina, Raquel Lucas-González, Manuel Viuda-Martos, Juana Fernández-López, José Ángel Pérez-Álvarez, Maria Martuscelli, Clemencia Chaves-López

**Affiliations:** a Bioscience and Technology for Food, Agriculture and Environment, University of Teramo, Via R. Balzarini 1, 64100, Teramo, Italy; b Grupo de Investigación Biotecnología, Facultad de Ingeniería, Universidad de San Buenaventura Cali, Carrera 122 # 6-65, 76001, Cali, Colombia; c IPOA Research Group, Agro-Food Technology Department, Higher Polytechnic School of Orihuela, Miguel Hernández University, CYTED- Healthy Meat. 119RT0568 “Productos Cárnicos más Saludables”, Orihuela, Alicante, Spain

**Keywords:** Cacao shell, Cacao pod husk, Epicatechin, Isoquercetin, Water holding capacity, Spectrum color

## Abstract

The cacao shell (CS) and cacao pod husk (CPH), two of the most promising high-fiber co-products of the cacao agro-industrial chain, were evaluated to determine their potential incorporation into food products. This research determined bioactive compounds and techno-functional properties of CS and CPH, and was evaluated the enzymatic inactivation by thermal treatments in CPH. We found that CS is rich in protein, lipids, dietary fiber (48.1 ± 0.3 g 100 g_dw_^−1^), and antioxidant molecules such as epicatechin (1.10 ± 0.02 mg g^−1^) and isoquercetin (1.04 ± 0.09 mg g^−1^). Moreover, in CS a positive effect of hydration mechanism occur; in fact, it was observed a reduction of Lightness (L∗) value and a remarkable color difference (ΔE∗,18.8 ± 0.7) (CIEL∗a∗b∗ color space), between hydrated and dry CS samples; so, it could be used as a potential natural colorant in foods. CPH resulted equally rich in dietary fiber (35.3–37.4%) and flavonoids (2.9 ± 0.1 mg RE g^−1^); in this co-product, the rapid enzymatic inactivation by thermal treatments was essential to obtain the highest antioxidant activity and polyphenols content; regarding the techno-functional properties, it was found that CPH flour had high hydration capacity, so CPH can use it as a replacement for emulsifiers or water holding additives while incorporating the fiber and abundantly found antioxidants.

## 1 Introduction

The growing interest of the population to consume nutritious foods of natural origin and generate an additional benefit in their health has sparked researchers' interest to know in-depth which biomolecules can provide these benefits, how they work, and in the king of foods in which they are present. Some research has demonstrated that many of these biomolecules are found in the non-edible parts of vegetables, so it is necessary to delve into their benefits and find a way to incorporate them into the diet. These incorporations can be indirectly through the isolation of biomolecules or directly by incorporating the co-products or by-products that contain them, always avoiding reducing the food's sensory characteristics.

*The food of the gods*, this is how chocolate was known in the Mayan culture due to its multiple benefits to promote health [1, 2], and treat diseases [3]. There is scientific evidence, that chocolate influences on bone health [4], cardiovascular health [5], potential neuromodulatory and neuroprotective [6], among others [1]. The chocolate used in elaborating multiple food products worldwide is made from the beans of *Theobroma cacao* L (cacao), a tree native from the Amazon basin (where it spread throughout the world) [7]. This tree grows only in tropical regions; the primary producers are Côte d’Ivoire, Ghana, Indonesia, Ecuador, Nigeria, Cameroon, and Brazil [8].

Cacao is one of the most important agricultural products at present. It is used as a raw material to obtain multiple products in the food industry worldwide. Its commercialization includes processed products represented for the cacao exporting countries around 50 billion dollars in 2019 [9]. Criollo (*Theobroma cacao* L cv Criollo) is a cultivar of Colombia and other Latin American countries, known for its high quality. It was declared by the International Cocoa Organization (ICCO) as “fine” and “flavor”. This cultivar represents between 7% and 10% of the world's production, and 76% of this production comes from Colombia, Ecuador, Venezuela, and Peru.

Cacao is generated by removing the beans from the cacao pods. Thus, there are four types of co-products: cacao pod husk (CPH), placenta, cacao mucilage, and cacao bean shells (CS). The CPH represent about 70%–75% of the whole fruit; CPH is an important source of fibrous materials, including lignin, cellulose, hemicellulose, and pectin [10, 11, 12] and a source of bioactive compounds [13]. Usually, the CPH is broken down in the cacao plantation, causing environmental problems [14], and it is an inoculum of fungal diseases in cacao crops like black pod rot [15].

The placenta that holds the grains inside the fruit is removed before the fermentation process. It favors the grains' germination, decreasing the fermentation index [16]; therefore, it is generally left on the plantation together with the CPH. Few studies demonstrate its potential as a natural source of nutrients., antioxidants, and bioactive compounds [17, 18].

The mucilage, also called cacao pulp juice or sweating, is a white mass surrounding cacao beans [19]. Right after the pod is opened and during the fermentation stage, this mass begins to free itself from the beans [20]; the microorganisms' action gradually eliminates it in seven days approx. In some cases, the fermentation is carried out on the soil, causing contamination of the site or nearby water sources [19]. In some regions, the farmers collecting the exudate generated (rich in sugars) in the first hours (with a low microbial load). It is used to elaborate on some liqueurs [18], jams [21], among other traditional food products.

The cacao shell is obtained after the roasting and grinding process to facilitate their separation of the nibs. [22]. The cacao shell has a high nutritional value [23], potential nutraceutical [24], and health benefits are attributed [25] mainly to the following bioactive compounds: phenolic compounds with high antioxidant capacity, dietary fiber, theobromine, and lipids similar to those of cacao butter in which palmitic, stearic and oleic acids are the most abundant [26].

The cacao waste valorization is a challenge since the pods once opened are very susceptible to oxidation, causing its deterioration. As was mentioned above, the evaluation of these cacao co-products properties is the first step to decide the type of food in which they can be incorporated to achieve the full use of their functional properties and the biomolecules they possess. Due to the benefits already mentioned, this work focuses on determining the bioactive compounds and techno-functional properties of the cacao shell and cacao pods husk (two of the most promising high-fiber co-products of the cacao agro-industrial chain). Additionally, the enzymatic inactivation by thermal treatments in CPH was evaluated.

## 2 Materials and methods

Criollo cocoa samples (Cuatrecasas 13377 (COL)) [27] were collected in February 2020 directly from a farm located in Valle del Cauca (Colombia), located in the western part of the country, 4°07′53.0″ N latitude, 76°13′30.9″ W longitude, altitude 975 masl. The cultivation of Creole cacao is associated with citrus cultivation. The area has an average temperature of 25 °C, with a maximum of 33 °C during the day and 21 °C at night, relative humidity of 65–75%, annual average rainfall of 1100 mm with two quarterly periods of alternate rains with two droughts. It is located within a tropical dry forest.

### 2.1 Cacao pod husk flour (CPHF)

**Figure 1:**
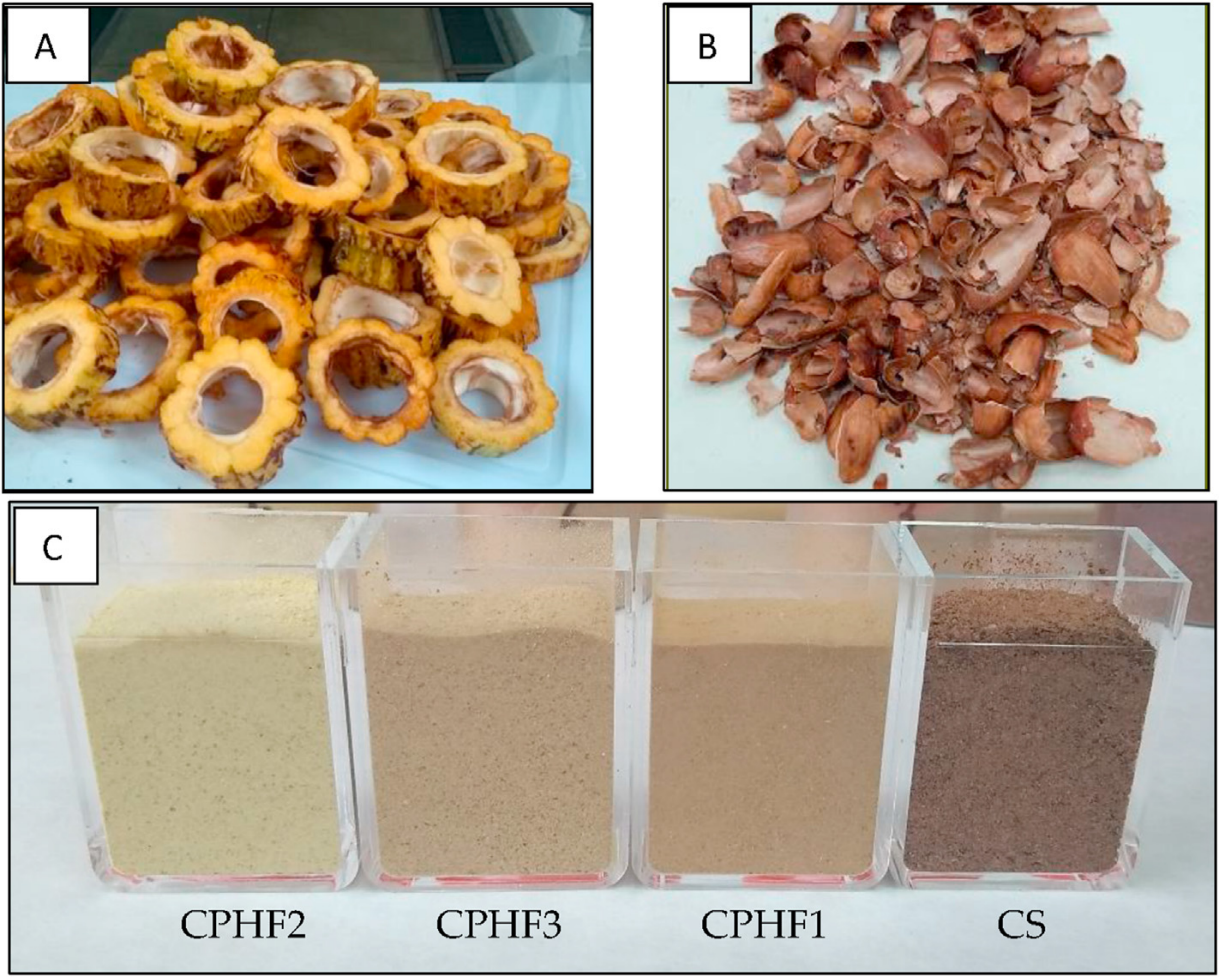
A. Cacao pods husk; B. Cacao shell; C. Samples dry and milled, CPHF2: Cacao pods husk flour treatment 2; CPHF3: Cacao pods husk flour treatment 3; CPHF1: Cacao pods husk flour treatment 1; CS: Cacao shell milled.

Fresh cacao fruits were collected and taken to the laboratory in dark bags where they were processed, and grains and mucilage were extracted. Cacao pods husk (CPH) was cut into 1 cm wide strips (Figure 1A) and subsequently divided in three batches that were subject to different treatments: CPHF1, control without heat treatment, froze immediately; CPHF2, samples were immediately placed in boiling water for 10 min to inactivate the enzymes; CPHF3, samples were placed at room temperature for 3 h (promoting enzymatic browning) and then they were placed in boiling water for 10 min to inactivate the enzymes.

Samples were allowed to cool and taken to freeze (-23 °C for 24 h) previously to undergo a freeze-drying process in a Liophilizer FreeZone 4.5 L (Labconco, Kansas, USA). Freeze-dried CPH were ground in a Hammer Mill M20 (IKA, Staufen, Germany), passed through a stainless-steel sieve (0.5 mm) to obtain the flour samples (here in after CPHF) (Figure 1C) and stored at 4 °C until used in the analyzes.

### 2.2 Cacao shell (CS)

The CS corresponds to the co-product of the roasting process of dry cacao beans at a temperature of 135 °C for 15 min (Figure 1B). The CS was ground in an impact grinding head MF 10.2 (IKA, Staufen, Germany) and passes through a stainless-steel sieve with circle shaped holes of 1 mm (Figure 1C).

### 2.3 Chemical, physico-chemical and physical analyses

The proximate composition was determined in CS and CPHF samples according to the following AOAC methods: lipid (AOAC 948.22), protein (AOAC 920.152), moisture (AOAC 925.45), ash (AOAC 923.03), total dietary fiber (TDF) (AOAC 985.29) [28], and the carbohydrate content were determined by calculating the percent remaining after all the other components have been measured (%carbohydrates = 100 - %moisture - %protein - %lipid - %TDF -%ash).

The pH was measured diluting the sample in distilled water in a ratio 1:1 for CS and 1:10 for CPHF with an electrode probe connected to a pHmeter (model 507 Crison, Spain). The water activity (aw) was measured at 25 °C using an electric hygrometer Novasina TH-500, (Novasina, Pfaeffikon, Switzerland). A colorimetric analysis was performed using a CM-700d Spectrophotometer (Konica Minolta, Osaka, Japan), depositing the samples in a rectangular cell of path length 20 mm (CM-A132) with the following settings (illuminant D_65_, observer 10°). The CIEL∗a∗b∗ color space was used [29]. The following color co-ordinates were determined: Lightness (L∗), red/green (+/-) co-ordinate (a∗), and (+/-) yellow/blue co-ordinate (b∗), chroma C∗ (Eq. (1)), hue angle H∗ (Eq. (2)), color difference ΔE∗ (Eq. (3)), and reflectance spectra (wavelengths 360nm–740nm) were determined. The browning index (BI) was calculated (Eqs. (4) and (5)) [30] and was used as an indicator of the effect of heat treatments on CPH. These parameters were calculated with follows equations:

(1) $$C^{*}=\sqrt{a^{*2}+b^{*2}},$$

(2) $${\text{H}}^{\text{∗}}=h_{ab}=\arctan\frac{b*}{a*}$$

(3) $$\Delta E^{*}=\sqrt{{\left(\Delta L^{*}\right)}^{2}+{\left(\Delta a^{*}\right)}^{2}{\left(\Delta b^{*}\right)}^{2}}$$

(4) $$BI=\frac{\left[100\left(x-0.31\right)\right]}{0.172}$$

(5) $$\text{Where: }x=\frac{\left(a^{*}+1.75L^{*}\right)}{\left(5.646L^{*}\right)+\left(a^{*}\right)-\left(3.012b^{*}\right)}$$

The reported values correspond to the average of nine measurements.

### 2.4 Polyphenols and antioxidants determination

#### 2.4.1 Extraction method

The methodology described by Fernández-López et al. [31], was followed with some modifications in order to extract polyphenols and antioxidant molecules in CS and CPHF samples.

In brief, 3.0 g the sample were mixed with 30 mL of methanol:water (80:20, v/v), vortex in a disperser a 20000 rpm for 3 min and then sonicated for 27 min at 20 °C. After centrifugation for 10 min, 8000 rpm at 4 °C, the supernatants were collected and the pellets were mixed with 30 mL of acetone:water (70:30, v/v) and the same procedure were repeated. The supernatants were combined and evaporated to dryness in a Rotavapor R-100 (Büchi Labortechnik AG - Flawil, Switzerland). The CS and CPHF extract were (content the polyphenol and antioxidant molecules) was resuspended in 5 mL of methanol, filtered through a 0.45 μm filter, and stored a -18 °C until use (see below).

#### 2.4.2 Total phenolic (TPC), total flavonoid (TFC), and total tannins (TTC) contents

Total phenolic content (TPC) was determined by the Folin-Ciocalteu method as described by Singleton and Rossi [32, 33]. The results were expressed as the standard mg gallic acid equivalents (GAE) g^−1^ of sample dry weight.

Total flavonoid content (TFC) was calculated by means of the method described by Blasa et al., [34]. In brief, 1.0 mL aliquot of an appropriately diluted polyphenolic extract was mixed with 0.3 mL 5% NaNO_2_; after 5 min, 0.3 mL 10% AlCl_3_; after 6 min, 2 mL 1M NaOH; and after 10 min the absorbance was determined at 510 nm. The results were expressed as the standard mg rutin equivalents (RE) g^−1^ of sample dry weight.

Total tanins content (TTC) was performed using the modified vanillin-HCl method in methanol described by Price et al., [35]. In brief, 1.0 mL aliquot of an appropriately diluted polyphenolic extract was mixed with 20 mL HCl 1% in methanol; after 20 min, were centrifuged at 2000 rpm for 4 min, the supernatant was separated, and mixed with 5 mL of vanillin solution (0.5% vanillin + 2% HCl in ethanol); and after 20 min, the absorbance was determined at 500 nm (Genesys 10uv spectrophotometer, Massachusetts, USA). The reaction was always maintained at 30 °C. The results were expressed as the standard mg catechin equivalents (CE) g^−1^ of sample dry weight.

#### 2.4.3 Polyphenols determination

The polyphenolic compounds were determined in the CS and CPHF methanolic extract by LC−MS/MS analysis in a Nexera XR system (Shimadzu, Tokyo, Japan) coupled to a 4500 Qtrap mass spectrometer (Sciex, Toronto, ON, Canada) equipped with a heated ESI source (V-source). The quantitation of the analytes was achieved with the standard addition method and the peak areas of the selected ions were defined using Sciex MultiQuant software according to Oliva et al., [36].

#### 2.4.4 Antioxidant activity

The antioxidant activity was determined how radical scavenging activity with 2,2-difenil-1-picrylhydrazyl (DPPH·) and 2,2′-azino-bis(3-ethylbenzthiazoline-6-sulfuric acid (ABTS^•+^) methods and expressed as Trolox Equivalent Antioxidant Capacity (TEAC); ferrous ion-chelating ability assay (FIC) and ferric reducing antioxidant power (FRAP). Results were calculated based on a calibration curve of Trolox and expressed as μmol of Trolox equivalent g^−1^ sample dry weight. A spectrophotometer (Genesys 10uv spectrophotometer, Massachusetts, USA) was used in all methods.

• DPPH· radical scavenging assay. The free radical scavenging activity of the methanolic extract of CS and CPHF was measured using the radical DPPH· according to the methodology described by Brand-Williams et al., [37]. Absorbance values were measured on a spectrophotometer at 517 nm.
• ABTS radical cation (ABTS^•+^) scavenging activity assay. The ABTS^•+^ scavenging activity assay on the methanolic extract of CS and CPHF was determined as described by Grande-Tovar et al., and Re et al., [33, 38]. Absorbance values were measured on a spectrophotometer at 734 nm.
• Ferric reducing antioxidant power (FRAP). This activity was determined in the methanolic extract of CS and CPHF by using the potassium ferricyanide-ferric chloride method [39]. Absorbance values were measured on a spectrophotometer at 700 nm.
• Ferrous ion-chelating ability assay (FIC). Ferrous ions (Fe^2+^) chelating activity of methanolic extract of CS and CPHF was measured by inhibiting the formation of Fe^2+^-ferrozine complex after treatment of methanolic extract with Fe^2+^, in accordance to the method of [40]. Absorbance values were measured on a spectrophotometer at 562 nm.

### 2.5 Techno-functional properties

The techno-functional properties of CS and CPHF were determined following as suggested by Fernández-López et al. [31], with some modifications.

The water holding capacity (WHC) was determined by adding 10 mL of water to 0.3 g of sample, homogenizing in a vortex for 1 min and leaving overnight to rest, the unabsorbed water was removed by centrifuge at 3000 rpm for 20 min. It was expressed as g of water retained per g of sample (w/w).

Oil holding capacity (OHC) was determined by adding 5.0 g of oil to 0.5 g of sample, homogenizing in a vortex for 1 min and leaving overnight to rest, the unabsorbed oil was removed by centrifuge at 3000 rpm for 20 min. It was expressed as g of oil retained per g of sample (w/w).

Swelling capacity (SWC) was determined by adding 10 mL of water to 0.5 g of sample in a graduated tube, homogenizing in a vortex for 1 min and leaving it at rest for 24 h. It was expressed as the volume change of the sample per g of sample (v/w).

Emulsion ability (EA) was determined by adding 1.0 g of sample to 50 mL of water, homogenizing in a disperser T25 digital Ultra Turrax (IKA, Staufen, Germany) at 8000 rpm for 2 min, followed by adding 50 mL of oil and homogenizing again for 1 min, 10 mL of the emulsion formed was deposited in a conical tube and centrifuged at 1500 rpm for 5 min, expressed as the percentage ratio between the volume of the emulsion layer formed and the total volume (% v/v). Emulsion stability (ES) was determined from the emulsion formed in the EA and heated to 80 °C for 30 min, cooled to room temperature and centrifuged at 1500 rpm for 5 min, expressed as the percentage ratio between the volume of the remaining emulsion layer and the initial volume of the emulsion (% v/v).

Foam Formation Capacity (FFC) was determined in a 500 mL beaker, 5.0 g of sample and 100 mL of water were deposited, homogenized in a disperser at 20000 rpm for 2 min, expressed as the percentage ratio between the foam formed and the volume initial of solution (% v/v). Foam Formation Stability (FFS) was expressed as the FFC but after 5 min of rest.

### 2.6 Statistical analysis

The one-way analysis of variance (ANOVA) was carried out to evaluate the statistical significance (P < 0.05) of the treatments. The means comparisons were made using Tukey HDS test (P ≤ 0.05). All data are presented as mean values ± standard deviation (SD). Analyzes were repeated three times unless indicated. The Statgraphics Centurion XVI program (Statgraphics Technologies, Inc. The Plains, Virginia, USA) was used for these statistical analyses.

## 3 Results

### 3.1 Proximate composition

In Table 1, bromatological parameters of the high-fiber co-products are showed. It is observed that this CS has lower total dietary fiber content, a higher lipid content, and similar protein content than that reported for other CS [41, 42]; these results may be dependent on the cacao variety and from the technology used to separate the shell from the grain [43]. The main protein contribution comes from the embryo, and the main lipid contribution comes from small grain fractions that, being lighter, are separated from the grains during the shell removal process.

**Table 1:** Proximate composition (g/100 g sample dry weight) and physico-chemical parameters of the investigated cacao shell (CS) and cacao pod husk flour (CPHF).

	Cacao shell (CS)	Cacao pod husk flour (CPHF)		
	CS	CPHF1	CPHF2	CPHF3
Protein (g/100 g _dw_)	15.2 ± 0.3 d	5.71 ± 0.02 b	4.85 ± 0.01 a	6.48 ± 0.04 c
Lipid (g/100 g _dw_)	15.1 ± 0.2 b	0.79 ± 0.03 a	0.8 ± 0.2 a	0.73 ± 0.02 a
Total dietary fiber (g/100 g _dw_)	48.1 ± 0.3 c	35.3 ± 0.9 a	37 ± 1 b	36.9 ± 0.8 b
Carbohydrates (g/100 g _dw_)	14.7 ± 0.7 b	49.3 ± 0.9 a	50 ± 1 a	49.0 ± 0.9 a
Moisture (g/100 g _dw_)	1.9 ± 0.3 b	1.1 ± 0.3 a	1.1 ± 0.2 a	2.3 ± 0.4 c
Ash (g/100 g _dw_)	7.0 ± 0.5 a	8.9 ± 0.3 b	7.3 ± 0.1 a	6.9 ± 0.4 a



pH	5.34 ± 0.02 a	5.61 ± 0.01 c	5.47 ± 0.03 b	5.56 ± 0.03 c
a_w_	0.59 ± 0.02 c	0.30 ± 0.02 a	0.32 ± 0.02 a	0.43 ± 0.03 b

Legend: Results are expressed as means ± standard deviations. Different letters in the same row indicate significant differences (P < 0.05).

In CPHF, the protein content of 4.85–6.48%, lipid content of 0.73–0.79%, and the total dietary fiber content of 35.3–37 % were found. The total dietary fiber reported by different authors varies widely (18.3%–59.0%); however, the protein and lipid values are within the reported ranges [13]. From the nutritional value, the protein contribution found can mean an added value to the different products; however, studies on the nutritional value and bioavailability of the amino acids are lacking. Although CPH has been proposed for any use in animal feed, more studies are required to achieve economically viable exploitation [13].

The soluble fiber content that corresponds to 30% of the total dietary fiber is mainly attributed to pectin [12]. The pectins contained in CPH are low methoxyl and highly acetylated [44], not require the presence of divalent cations to form gels contrary to other low methoxyl pectins. They can form a gel in an acid medium [45]; this is advantageous for its use in the pharmaceutical and food industry. The pectin extraction method is important to achieve a higher methoxyl degree; modern extraction methods with supercritical water have an advantage over conventional methods that use mineral acids [46] or organic acid [47] for their extraction [12].

The total dietary fiber, protein, and lipid content of CS are higher than CPHF. In general, the total dietary fiber contents found in CS and CPHF are slightly low compared to other fibers derived from agro-industrial co-products and which used as dietary fiber source, among them Mexican lime peel [48], Chia [49], orange waste, carrot pomace, potato peels, and green pea peels [50]. However, in CS, the protein and lipid content are high, so that its use as an alternative ingredient in foods becomes more important for a more significant nutritional contribution. Ash content is consistent with most vegetables.

The effect of the treatments on CPH showed a different impact on the proximal composition; the protein content concerning CPHF1 decreased in CPHF2 and increased in CPHF3 (P < 0.05), while the fiber content increased. A significant decrease was also observed in ash content (P < 0.05) and lipid (non-significant) content. These changes are mainly attributable to the solubilization of small amounts of lipids, minerals, and soluble fiber.

### 3.2 Physico-chemical properties (a_w_ and pH)

The drying treatments on the fibers are consistent with the low moisture content and water activity found, which can mean a low probability of proliferation of microorganisms that deteriorate the fibers. The pH found in the fibers is slightly acidic, in CS due to traces of organic acids originated during the fermentation and drying process of the beans [51], and in CPHF due to the ripening process. The effect of the treatments on CPH showed a significant decrease (P < 0.05) in pH (Table 1). The pH decrease is attributed to the protection of organic acids that occurs when the enzymatic action is inhibited by treatments [52].

### 3.3 Color

The CS color parameter is shown in Table 2. The L∗, a∗, and b∗ color co-ordinates were similar to those reported for cacao bean powder [53]. In a related way to cacao powder, the color of CS does not depend on a single molecule, it is a complex interaction of polyphenols and anthocyanins that during fermentation, drying, and roasting processes lead to complex structures such as phlobaphene, which contribute to the characteristic brown color of roasted beans [54]. Also, oxidation and polymerization of polyphenols, degradation of proteins, and Maillard reactions with the consequent formation of high molecular weight melanoidins [55].

**Table 2:** Color parameters of co-products of the cacao agro-industrial chain.

Sample	L∗	a∗	b∗	C∗	H∗	ΔE∗	BI
Dry							
CS	51.49 ± 0.04 d	10.62 ± 0.01 e	19.32 ± 0.01 c	22.05 ± 0.01 d	61.21 ± 0.03 c		60.66 ± 0.09 e
CPHF1	65.3 ± 0.2 f	7.81 ± 0.06 cd	19.9 ± 0.2 d	21.4 ± 0.2 c	68.56 ± 0.01 e		44.1 ± 0.5 b
CPHF2	78.9 ± 0.9 h	4.0 ± 0.3 a	25.2 ± 0.3 f	25.5 ± 0.4 f	80.9 ± 0.4 h	15.1 ± 0.7 †	41 ± 1 a
CPHF3	68.82 ± 0.06 g	6.90 ± 0.09 b	20.2 ± 0.2 d	21.3 ± 0.2 c	71.04 ± 0.06 f	3.67 ± 0.04 †	41.0 ± 0.5 a
Hydrated							
CS	35.4 ± 0.5 b	8.1 ± 0.3 d	10.0 ± 0.4 a	12.9 ± 0.5 a	51.1 ± 0.2 a	18.8 ± 0.7 a‡	49.0 ± 1 c
CPHF1	33.6 ± 0.2 a	7.5 ± 0.3 bc	10.89 ± 0.02 a	13.3 ± 0.1 a	55 ± 1 b	32.9 ± 0.2 d‡	54.2 ± 0.8 d
CPHF2	53.3 ± 0.5 e	4.6 ± 0.5 a	23 ± 1 e	23.5 ± 1.3 e	78.8 ± 0.7 g	25.7 ± 0.6 b‡	61 ± 4 e
CPFH3	40.2 ± 0.8 c	8.1 ± 0.3 d	15.3 ± 0.7 b	17.3 ± 0.7 b	62.2 ± 0.1 d	29.1 ± 0.9 c‡	61 ± 2 e

Abbreviations: L∗, lightness; a∗, red/green coordinate; b∗, yellow/blue coordinate; C∗, Chrome; H∗, hue; ΔE∗, Color differences; BI, Browing index. † symbol indicates values with respect to CPHF1, ‡ symbol indicates values with respect to its dry equivalent. Results are expressed as means ± standard deviations. Different letters in the same column indicate significant differences (P < 0.05).

The dried CS showed an L∗ value of 51.49 ± 0.04, which decreased to 35.4 ± 0.5 when subjected to hydration; this behavior can be due to several mechanisms. Thus, the first hypothesis is microstructural; the water “moves” inside the macromolecules, changing their ultrastructure and diminishing the amount of free water free on the surface, causing the reduction of L∗. The second one is related to the migration of natural colorants from CS. These compounds move to the surface, causing a decrease of L∗. In this process, the visually brown color (appreciable to the human eye, ΔE∗ > 3) is accentuated, showing an ΔE∗ of 18.8 ± 0.7. Thus, CS can be used as a potential natural colorant, mainly due to the global trend to reduce artificial additives as colorants [56], with the plus that is from cacao, a globally well-appreciated food.

The color of CPH depends on the content of proanthocyanidins present, like other polyphenolic compounds such as catechin, epicatechin, and isoquercetin, which accumulate mainly in the exocarp and give it different colors depending on the variety of cacao [13]. The mesocarp and endocarp are light yellows in color; however, they can undergo rapid browning when exposed to air once opened.

The effect of the thermal treatments was observed mainly in an increment in the L∗ and a∗ co-ordinates concerning CPHF1 (control) (Table 2), which indicates that the treatments had a positive impact by avoiding a decrease in lightness, a parameter directly related to the BI, as well as a positive effect in avoiding the loss of the yellow color attributed to the pigments present in the exocarp in this cacao variety.

The BI found for CPHF1 was 44.1 ± 0.5, which decreased significantly with heat treatments (P < 0.05), presenting values of 41 ± 1 and 41.0 ± 0.5 for CPHF2 and CPHF3, respectively, showed no statistical difference between them (P > 0.05). This decrease in BI is directly related to decreased polyphenol oxidase activity [30] and a lesser extent with the waiting time to do it.

When hydrating the CPHF, appreciable changes are observed (ΔE∗ > 3), mainly attributed to the L∗ parameter decrease. As in CS, it is a measure of its ability to absorb water and reflect less light.

**Figure 2:**
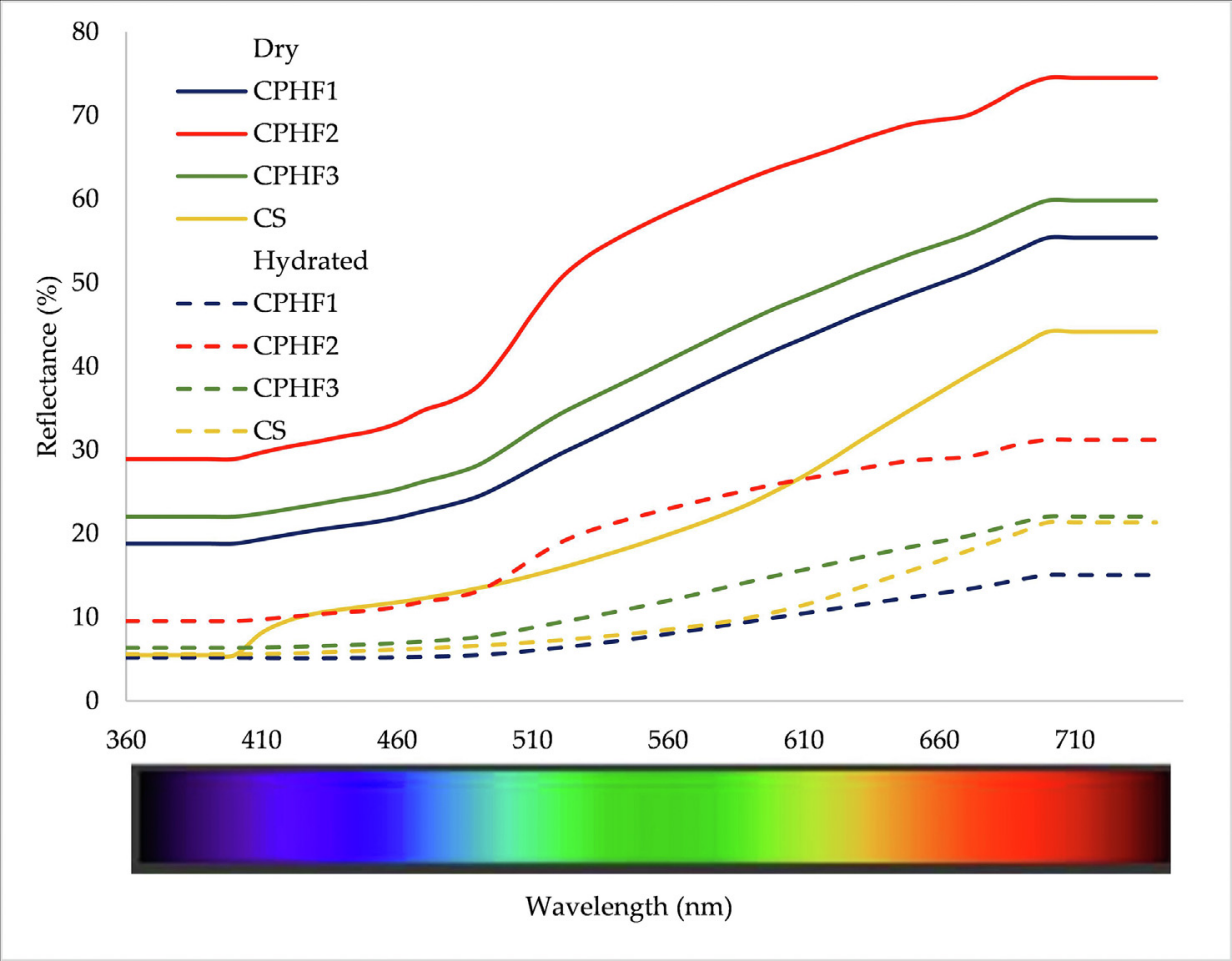
Reflectance spectra (360–740 nm) of the cacao shell (CS) and cacao pod husk flour for the three treatments (CPHF1, CPHF2, and CPHF3). Solid lines indicate measurement of dry co-products and dotted lines indicate measurement of hydrated co-products.

Figure 2 presents the reflectance spectrum (360–740 nm) obtained from the CS and CPHF. A pronounced increase was observed in CS above 600 nm, indicating a considerable contribution of phenolic compounds and melanoidins (red pigments). In comparison, in CPHF, there was a marked increase above 500 nm, indicating a contribution of photosynthetic pigments (green, yellow, and orange pigments) and phenolic compounds. In the CPHF treatments (CPHF2 and CPHF3) a decrease in reflectance was caused by the oxidation and browning processes that occurred.

When water was added to the fibers, the spectrum decreased (P < 0.05). This behavior is similar when water is added to the paprika [57]. Usually, when water is incorporated in fibers the reflection spectrum is increased because solid particles with water on the surface (adsorbed) reflect more light (specular reflectance), while in humidified particles where the water is absorbed in the matrix is reduced reflection (diffuse reflectance).

### 3.4 Polyphenols

The results by LC−MS/MS analysis show three mainly polyphenolic compounds (catechin, epicatechin, and isoquercetin) (Table 3). Known for their antioxidant activity, it represents 32% of the polyphenols found in the CS and 9–14 % in CPHF, possibly main responsible for the antioxidant activity found. This, supported by the linear correlation analysis between polyphenolic molecules and antioxidant activity, which showed that catechin and epicatechin present a linear relationship with antioxidant activity FRAP (0.77), ABTS (0.65), DPPH (0.70), and TPC (0.69). At the same time, isoquercetin is related to TTC (0.98). Although the fermentation, drying, and roasting processes through which the CS goes through tend to decrease the number of polyphenols significantly, the values found are similar to cacao samples reported for cacao beans [58].

**Table 3:** Polyphenols and antioxidant activity in co-products of the cacao agro-industrial chain.

Compound	CS	CPHF1	CPHF2	CPHF3
Catechin (mg g^−1^)	0.87 ± 0.02 c	0.32 ± 0.03 b	0.96 ± 0.02 d	0.20 ± 0.03 a
Epicatechin (mg g^−1^)	1.10 ± 0.02 c	0.41 ± 0.05 b	1.22 ± 0.02 d	0.25 ± 0.04 a
Isoquercetin (mg g^−1^)	1.04 ± 0.09 c	0.20 ± 0.08 b	0.09 ± 0.09 a	0.09 ± 0.07 a
TPC (mg GAE g^−1^)	9.53 ± 0.03 c	8.44 ± 0.04 b	16.6 ± 0.4 d	5.4 ± 0.4 a
TFC (mg RE g^−1^)	3.21 ± 0.08 b	2.9 ± 0.1 b	8.4 ± 0.3 c	1.8 ± 0.1 a
TTC (mg CE g^−1^)	1.30 ± 0.04 d	0.19 ± 0.01 b	0.25 ± 0.02 c	0.12 ± 0.03 a



TEAC (ABTS) (μmol TE g^−1^)	65 ± 3 c	55 ± 1 b	140 ± 4 d	32.9 ± 0.5 a
TEAC (DPPH·) (μmol TE g^−1^)	71 ± 1 c	58 ± 4 b	133 ± 1 d	36 ± 1 a
FRAP (μmol TE g^−1^)	106 ± 4 c	77 ± 4 b	181 ± 6 d	53 ± 2 a
FIC (μmol TE g^−1^)	4.7 ± 0.1 b	9.3 ± 0.1 c	4.0 ± 0.5 a	4.5 ± 0.1 ab

*Legend:* Results are expressed as sample dry weight. Results are expressed as means ± standard deviations. Different letters in the same row indicate significant differences (P < 0.05). Abbreviation: GAE, gallic acid equivalent; RE, rutin equivalent; CE, catechin equivalent; TE, Trolox equivalent.

The effect of pretreatments on CPH showed that the rapid inactivation of enzymes protected the polyphenolic compounds; in CPHF2, catechin and epicatechin presented the highest values (3 times greater than the control) and even higher than CS. Valadez-Carmona et al. [59], observed that drying with lyophilization shows better results than drying with hot air or microwaves; our results show that the direct lyophilization process can be improved by almost 100% (8.44 ± 0.04 to 16.6 ± 0.4 mg GAE g^−1^) with enzymatic inactivation heat pretreatments.

Active enzymes may cause a greater decrease in polyphenols than heat treatments. At present, there are no studies on the optimal temperature, the type or the concentration of chemical inhibitors, the time of heat treatment to decrease the enzymatic activity that affects the polyphenols present in the pod. Some studies carried out on cocoa beans show that temperatures of 96 °C for 6.4 min can greatly decrease the enzymatic activity and obtain the highest content of polyphenols [60].

The total phenol content (TPC) in CS is higher than those reported by other authors in Ecuador (1.5 mg GAE g^−1^ sample) [42], but less than those reported in Malaysia (16.3–41.4 mg GAE g^−1^ sample) [61]. Criollo cultivars are rich in polyphenols [58]; small variations in these compounds are attributable to differences in the fermentation, drying, and roasting [43]. Similarly, CPHF1 to CPHF3 presented higher values than those reported in Ecuador (3.6 mg GAE g^−1^ sample); but lower than those reported in Mexico (18.93 mg GAE g^−1^ sample) [59] and Malaysia (19.6–49.5 mg GAE g^−1^ sample) [61].

The flavonoids in CS and CPHF1 to CPHF3 presented lower values than those reported in Malaysia (8–25 and 4.5–22.4 mg RE g^−1^ sample), respectively [61]. However, the amounts found are of great interest because these flavonoids are similar to that of cocoa beans reported in Colombia (2.5–11.3 mg RE g^−1^ sample) [58].

The Tannins of CS and CPHF1 to CPHF3 show lower values than reported by Lecumberri et al., (10.4 mg g^−1^ sample) and Oduro et al., (2.5 mg g^−1^ sample) [41, 62]. Lower amounts of tannins are advantageous when making products from by-products since they will be marked to a lesser extent by the bitter flavors attributed to these compounds.

In general, polyphenolic compounds' content was higher in CPHF2, indicating that the rapid inactivating of enzymes can protect these compounds. CPHF3 (3-hour wait before inactivation) and CPHF1 (direct freezing without inactivating enzymes) showed a significant decrease (P < 0.05) of the polyphenolic compounds compared to CPHF2. Although the freezing process in CPHF1 could inactivate enzymes, the time used to lower the temperature to conditions of little enzymatic activity (not determined) evidences a rapid reduction of the compounds. In CPHF3, although the enzymes were thermally inactivated after three hours, lower values were obtained in TPC and TFC than those obtained in CPHF1, which had immediate cooling, which suggests that the enzymes act quickly at room temperature and decrease their activity with decreasing temperature. As lyophilization is one of the best techniques to preserve polyphenolic compounds [59], the pre-treatment applied in CPHF2 improves said efficiency.

### 3.5 Antioxidant activity

The evaluation of the antioxidant activity is a parameter of difficult comparison due to the different processing and extraction methods and data reporting ways [13].

In CS, the values found in TEAC (Table 3) are higher than reported in CS from Ecuador (DPPH· 3.81–4.05 μmol TE g^−1^; ABTS 4.56–4.45 μmol TE g^−1^) [42] and from a Spanish industry that processes cacao (7.73 μmol TE g^−1^) [41]. However, it's are lower than reported from an Italian industry that processes cacao from Ecuador (DPPH· 204.7–218.3 μmol TE g^−1^) [26] and from the Spanish industry that processes cacao (ABTS 134.2 μmol TE g^−1^) [63], which improved extraction methods. The same trend was found in FRAP, which is higher than (72.32 μmol TE g^−1^ and 1.51–1.78 μmol TE g^−1^) reported by Lecumberri et al., and Martínez et al. [41, 42], respectively. But less when extraction methods are improved (141.8 μmol TE g^−1^) [63]. The Fe^2+^-chelating ability is the first time that value is reported in CS.

In CPHF1, the values found in TEAC are higher that reported in CPH from Ecuador (ABTS 37.97–42.93 μmol TE g^−1^; DPPH· 33.07–33.93 μmol TE g^−1^) [42], from Côte d’Ivoire (ABTS 51.87 μmol TE g^−1^) [64], and from Mexico (ABTS 30.6 μmol TEAC g^−1^; DPPH· 15.1 μmol TEAC g^−1^) [59]. However, they are lower than reported in another Colombia region (ABTS 116,0–229,6 μmol TE g^−1^) [65]. The values found in FRAP are lower than reported from Côte d’Ivoire (129.5 μmol TE g^−1^) [64] and (136.6–169.0 μmol TE g^−1^) [65], and higher than reported from Ecuador (4.49–4.69 μmol TE g^−1^) [42].

In CPHF, differences were found (P < 0.05) between treatments, the antioxidant activity (TEAC and FRAP) was higher in CPHF2, conserving the same trend found between treatments for the content of polyphenols, which assumes that the antioxidant activity in CPHF is directly related to the polyphenol content present, the CPHF2 treatment better preserved the polyphenols and therefore the antioxidant activity is favored.

Also, this is the first time that Fe^2+^-chelating ability value is reported in CPHF. This capacity is directly related to the ability to chelate iron. In cacao, this capacity may be due to phytochelatins and metallothioneins, which protect the cacao plant from the adsorption of Cadmium present in some soils where it is cultivated [66]; this should be studied in greater depth, although the consumption of these biomolecules does not represent a threat to the consumers' health, the same care should be taken as with chocolate when the metal is present in the soil and the conditions are conducive to its absorption and bioaccumulation in the plant [67].

In CPHF1, the FIC value was higher (P < 0.05) than CPHF2 and CPHF3, it is possible that the temperature applied in these two treatments denatured some molecules (phytochelatins and metallothioneins) responsible for this chelating activity, therefore a lower FIC value (Table 3).

In general, the antioxidant activity values in CS and CPHF are also higher than those reported in fortified flours, bread, and toasted bread [68], which indicates that are co-product rich in antioxidant compounds derived mainly from the polyphenols it possesses.

### 3.6 Techno-functional properties

#### 3.6.1 Water holding capacity (WHC), oil holding capacity (OHC) and swelling capacity (SWC)

Results for the water holding capacity (WHC), oil holding capacity (OHC) and swelling capacity (SWC) of the CS are presented in Table 4.

**Table 4:** Technological properties of co-products of the cacao agro-industrial chain.

Parameter	CS	CPHF1	CPHF2	CPHF3
WHC (g g^−1^)	4.6 ± 0.3 a	27.2 ± 0.5 b	29 ± 2 c	29 ± 1 c
OHC (g g^−1^)	1.30 ± 0.07 a	1.81 ± 0.03 b	1.99 ± 0.02 c	2.22 ± 0.02 d
SWC (mL g^−1^)	5.2 ± 0.5 a	17.8 ± 0.5 d	15 ± 1 c	12.4 ± 0.5 b
EA (% v/v)	45 ± 1 a	81.1 ± 0.3 d	61.0 ± 0.6 b	76.1 ± 0.3 c
ES (% v/v)	95.9 ± 0.2 b	95 ± 1 b	73 ± 4 a	72.7 ± 0.9 a
FFC (% v/v)	73 ± 2 d	27.27 ± 0.01 c	22.73 ± 0.02 a	22.73 ± 0.01 a
FFS (% v/v)	28 ± 3 c	22 ± 4 b	18.18 ± 0.04 a	18.18 ± 0.01 a

Abbreviations: WHC, water holding capacity; OHC, Oil holding capacity; SWC, Swelling capacity; EA, Emulsion ability; ES, Emulsion stability; FFC, Foam Formation Capacity; FFS, Foam Formation Stability. Different letters in the same row indicate significant differences (P < 0.05).

Data showed that the hydration capacity of the CS is similar to that reported by other authors in the same matrix [41, 42], but lower if they are compared to other agricultural co-products and which used as a dietary fiber source, among them Mexican lime peel [48], orange waste, carrot pomace, potato peels, and green pea peels [50]. However, the application or use also depends on other important characteristics, the nature of the product to which it is wanted to incorporate, the contribution of other nutritional or functional characteristics, the direct impact on the texture properties, or the possibility of improving those properties in advance by physical-chemical treatments as it has been demonstrated with insoluble fiber sources such as soybean fiber [69] or possibly *in situ* within the product processing treatments.

In CPHF1, CPHF2, and CPHF3, the parameters WHC, OHC, and SWC, are higher than reported by Yapo et al. [64], (WHC 6.49 g g^−1^, OHC 1.92 g g^−1^, and SWC 7.31 mL g^−1^) and Martínez et al. [42], (WHC 5.86 g g^−1^, OHC 1.20 g g^−1^, and SWC 5.81 mL g^−1^). This can be attributed to the drying method carried out; these values correspond to samples dried with convective heat (40–45 °C and 60 °C, respectively), which can cause cell collapse and loss of turgor, reflecting in lower properties derived from the holding capacity, compared to the lyophilized samples [59].

The differences found (P < 0.05) in CPHF treatments show that WHC and OHC were higher in CPHF2 and CPHF3, while SWC decreased. It is possible that the heating before the lyophilization process allowed the opening of cavities favoring the retention capacity, but at the same time, it deteriorated the structure and prevented it from swelling completely. This behavior was more pronounced in the CPHF3 treatment; it is possible that the time elapsed before the heat treatment (three hours) not only increased the browning index due to enzymatic activity but also allowed expansin-like proteins to continue with the degradation process of the cell wall that occurs during maturation [70]. Additionally, leaving exposed polar groups which would increase the OHC and debit the structure, further decreasing the swelling capacity.

In CPHF, these properties were higher compared to CS and other fibers derived from agricultural co-products and which used as a dietary fiber source, among them Mexican lime peel [48], orange waste, carrot pomace, potato peels, and green pea peels [50], which gives it an advantage for its use over other fibers.

#### 3.6.2 Emulsion ability (EA) and stability (ES)

Emulsifying ability (EA) is a molecule's ability to act as an agent that facilitates solubilization or dispersion of two immiscible liquids, and emulsion stability (EE) is the ability to maintain the integrity of an emulsion. All CPHF samples showed higher EA values than CS (P < 0.05), suggesting that the roasting process has a negative impact on this property. In the case of CPHF samples, heat treatment also decreased their EA. However, all of them reached values higher than 50%, which is considered desirable, suggesting good potential to act as an emulsifier in food formulation [71]. It is possible that the loss of lipids or the denaturation of proteins by heating influences this property. Furthermore, CPHF1 and CS showed the highest (P < 0.05) values for ES (higher than 90%, without differences between them), suggesting that both samples can be used to stabilize an emulsion once formed [72]. CPHF2 and CPHF3 samples (both of them heat-treaded) showed the lowest (P < 0.05) ES values (without differences between them). Because this data, it could be said that CPHF1 is the better sample to be used both as an emulsifier (the highest value for EA) and as an emulsion stabilizer (the highest value for ES); these properties could be required for emulsified food with long shelf-life (long emulsion stability). Additionally, the formation and stabilization of emulsions depend on phenomena such as surface tension, molar mass, and surface charge of the emulsifier. In this case, the fiber composition incorporates soluble and insoluble fibers, proteins, and lipids, which can act differently in the stabilization or formation of emulsions; therefore, this capacity must be tested in different food matrices, especially in meat products.

#### 3.6.3 Foam formation capacity (FFC) and stability (FFS)

In CS, FFC was high (72.73%), which indicates that it can form a foam or a high surfactant capacity, possibly related to a low net charge and adequate balance between hydrophobicity and hydrophilicity. However, the formed foam is unstable (27.27% FFS). While CPHF presented low percentages, which indicates that it is not suitable for foam formation or stabilization, it does not decrease surface tension. Additionally, the heat treatments decreased this property, indicating a perturb in the balance between hydrophobicity and hydrophilicity. Specific studies should be carried on to describe these two behaviors.

### 3.7 Potential incorporation into food products

#### 3.7.1 Cacao pod husk

According to the information obtained, the use of CPH in food can be oriented in the first place in the increase of dietary fiber in processed foods that lack fiber, complementing the diet. Some studies have shown that CPH has the natural benefits of fiber consumption, such as obesity and cardiovascular risk improvement [73, 74]. However, it is necessary to bear in mind that due to its techno-functional properties, it can easily change the texture of the food to which it is added [75]. The CPH can be creating new connections between the components of the matrix [76], in addition to competing for the water available in the feed due to the high water holding capacity and swelling capacity of CPHF. This makes it necessary to reformulate the products, totally or partially substituting an ingredient within the formulation.

Although the OHC for CPH is low, it has high EA and ES, so it can be oriented to products such as comminuted meat products (meat emulsions such as sausages), in which it can affect the sticking, gelatinization properties, and granular morphology of the matrix that contains it [77], could replace the starch (total o partially) that is the accepted ingredient to stabilize this type of products. In dairy derivatives such as yogurt, the use of CPH can improve firmness and rheology [78] due to the presence of low-methoxyl pectins [79].

Attempts have been made to integrate CPH into bakery products with little benefit [80]. In high concentrations, an increase in the bread's hardness can occur due to the high concentration of fiber, greater cohesion force generated due to dehydration during baking, and restricted gas expansion. Additionally, there is a darkening due to oxidation reactions in the bread crust due to air contact with the pigments and polyphenols of CPH and the increase in temperature.

Bioactive compounds can play an essential role in technological applications; when process products are subjected to high temperatures, enzymatic browning and other oxidation reactions favored under certain conditions can reduce the nutritional value of the food they incorporate. It is clear that the use of CPH is important for the direct contribution of bioactive compounds, mainly pigments, and polyphenols that provide a high antioxidant capacity, but the technological properties of CPH relegate it.

#### 3.7.2 Cacao shell

Due to the high amount of polyphenols and antioxidants that CS has, its use in food can be aimed at obtaining food additives or flavor enhancers. The small cocoa contribution gives this co-product a particular characteristic since it preserves many cocoa properties such as aroma, taste, and color. In this sense, some patents have already been obtained, and its effectiveness as a lipid antioxidant agent has been demonstrated [23, 81].

The incorporation of CS in some food products can have a dual purpose, the nutritional contribution of protein and lipids and the functional contribution of dietary fiber, polyphenol compounds, and antioxidants. However, the aroma and taste of chocolate may mean that its use is restricted to products already known to have such a flavor. Its incorporation in cookies [82], wheat bread [83], sponge cake [84], and muffins [85] have had success due to the chocolate flavor that it incorporates, due to the increase in fiber in the products and because it allows the incorporation and replacement of part of the fat added in the preparation, in addition to the antioxidants and polyphenols that are preserved very well after of cooking. This opens the way for its incorporation into other baked goods.

The use of CS in infusions is the most widespread in some countries; some studies showed biomolecules' migrate to the drink [86]. However, studies of their nutritional profile have not yet been carried out.

The information obtained can be used by the food industries for their incorporation into a wide range of products, for example: in healthy snacks given their high demand, making it possible to improve the nutritional properties of those products that are dense in calories and nutritionally very poor, making it a nutritional fortification agent [87]; in meat emulsions due to the high lipid content and its ability to stabilize emulsions, it is possible to incorporate it into meat products as a fat replacement, and additionally, the incorporation of fiber would be significant.

The introduction of CS and CPHF in food must encompass different strategies to achieve successful incorporation within the food matrix, whose final jury will be the consumers. The use must be planned as an integral part of the chain, allowing implementation techniques that ensure quality and safety for food use. Even though there are difficulties for its immediate implementation [88], the food industries can use technology to overcome them.

### 3.8 Potential in indirect incorporation through its use in animal feed

In this field, the research should be focused on improving the quality of meat and eggs. The use of CPHF in animal feed has focused mainly on the contribution of proteins and carbohydrates to the diet, leaving aside nutritional quality improvement by incorporating their antioxidant compounds. Some obstacles have been found for its use in high proportions (>20% of the diet) in monogastric due to the high fiber content, high water holding capacity, and swelling capacity of CPH, which together can lead to reduced digestibility and an increase in intestinal viscosity that manifests itself with weight loss. Studies have been carried out with promising results in growing pigs [89] and fish [90], and others with less acceptance in broiler chick [91].

The CS and CPH use to incorporate pigments and antioxidants into the poultry diet may be important in the quality of meat and eggs, as demonstrated with other co-products [92]. Develop the full potential of CPH for animal feed may be possible with pretreatments that allow enhanced antioxidant activities, nutritional qualities, and digestibility. In this sense, the use of enzymes or solid-state fermentation is promising [93].

Studies on the use of CS in animal feed are still incipient. The main obstacle is the high concentration of theobromine that tends to reduce some species of animals' weight. In chickens, it resulted in a decrease in the average daily feed intake and egg production [94]. However, the theobromine removal can be done by hot water extraction treatments with good results in digestibility [95]. Further studies are required to evaluate the detriment of antioxidant compounds in removing theobromine from CS.

## 4 Conclusions

The knowledge of the physicochemical characteristics, the techno-functional properties, and the content of bioactive compounds that the organic cacao shell and pod husk possess is the first step to propose using these co-products to industrial scale.

The techno-functional properties found in the cacao shell, such as the content of proteins, lipids, dietary fiber, and antioxidant molecules such as epicatechin and isoquercetin, show great potential for incorporation into conventional foods products or for the development of new food products. This incorporation could improve consumers' health and well-being, with the advantage that the cacao shell has many of the sensory characteristics of cacao, that could be well accepted by consumers.

In cacao pod husk, it is essential to highlight that the rapid inactivation of enzymes by thermal treatments is critical to preserve the highest antioxidant activity and polyphenols content. From an industrial point of view, the high amount of dietary fiber and its water holding capacity suggest that it can be used as a good substitute for emulsifiers or water retainers replacement, incorporating at the same time the fiber and antioxidant properties into new food products.

This strategy of valorization of cocoa co-products is part of the circular bioeconomy and can be applied by local farmers. However, production costs need to be assessed and require future research to ensure sustainable harvest where industry or government involvement is needed. Although it is still necessary to evaluate other alternatives that may have the same or better results with a lower cost, impacting the first step of the agro-industrial chain generates a new income opportunity for the primary sector.

## Declarations

### Author contribution statement

Johannes Delgado-Ospina, Manuel Viuda-Martos: Conceived and designed the experiments; Performed the experiments; Analyzed and interpreted the data; Contributed reagents, materials, analysis tools or data; Wrote the paper.

Raquel Lucas-González: Performed the experiments.

Juana Fernández-López, José Ángel Pérez-Álvarez, Clemencia Chaves-López: Conceived and designed the experiments; Analyzed and interpreted the data; Contributed reagents, materials, analysis tools or data; Wrote the paper.

Maria Martuscelli: Performed the experiments; Analyzed and interpreted the data; Wrote the paper.

### Funding statement

This work was supported by Colciencias, Patrimonio Autónomo Fondo Nacional de Financiamiento para la Ciencia, la Tecnología y la Innovación Francisco José de Caldas. (C. 808–2018. Agreement 240–2019. Number 123280864259).

### Data availability statement

Data will be made available on request.

### Declaration of interests statement

The authors declare no conflict of interest.

### Additional information

No additional information is available for this paper.
